# Low Density Wood Particleboards Bonded with Starch Foam—Study of Production Process Conditions

**DOI:** 10.3390/ma12121975

**Published:** 2019-06-19

**Authors:** Sandra Monteiro, Jorge Martins, Fernão D. Magalhães, Luísa Carvalho

**Affiliations:** 1 LEPABE—Laboratory for Process Engineering, Environment, Biotechnology and Energy, Faculty of Engineering, University of Porto, Rua Dr. Roberto Frias, 4200-465 Porto, Portugal; sandram@fe.up.pt (S.M.); jmmartins@estgv.ipv.pt (J.M.); fdmaglh@fe.up.pt (F.D.M.); 2 DEMAD—Department of Wood Engineering, Polytechnic Institute of Viseu, Campus Politécnico de Repeses, 3504-510 Viseu, Portugal

**Keywords:** pressing process, pressing parameters, lightweight, particleboard, sour cassava starch, foam

## Abstract

It has been shown that wood particleboards bonded with sour cassava starch can display low density combined with good physico-mechanical performance, thanks to starch being able to produce a strong foam that fills the interparticular space. Here we optimize the pressing conditions for the production of these panels. The procedure involved hot-plate pressing in two stages: (1) lowering the top platen to a specified thickness for a duration designated as pressing time, followed by (2) raising the top platen to allow panel expansion for a duration designated as hold time. The parameters studied were the pressing time (10 to 150 s), the hold time (290 to 890 s), and the top platen temperature (80 to 190 °C). The hold time and pressing time showed to be crucial parameters. The best operating conditions corresponded to 600 s of press cycle time, comprising 60 s of pressing time and 540 s of hold time. The top platen temperature used was 190 °C. The particleboards produced had a density of 405 kg·m^−3^, an internal bond strength of 0.44 N·mm^−2^, and a thickness swelling of 13.2%. This can be considered as very good performance, taking into account the panels’ low density.

## 1. Introduction

Particleboards are composites made of wood particles (wood flakes, chips, shavings, saw-dust and similar) and/or other lignocellulose materials in particle form (flax shives, hemp shives, bagasse fragments and similar), with the addition of an adhesive, bonded together with an adhesive system under pressure and heat (EN 309). The most commonly used adhesives are formaldehyde-based resins, mainly urea-formaldehyde resins. Particleboard densities are usually in the range 600 to 750 kg·m^−3^ [1]. Particleboards with density below 600 kg·m^−3^ are designated as lightweight (CEN/TS 16368). That makes their application in furniture industry easier where low weight is required to facilitate transportation and assembly by the customer. Several strategies are available to produce lightweight particleboards, such as lower compaction of the wood mat, use of light wood species, use of sandwich panels with foam core (made of polyurethane or polystyrene foam) or cardboard-based honeycomb core, and production of extruded particleboards containing longitudinal tubular hollow spaces. However, density reduction always has a negative impact on mechanical resistance, in addition to other problems such as difficulty in surface finishing and post-forming [2].

Currently, biosourced and biodegradable materials assume great industrial importance due to environmental issues, in particular regarding replacement of petroleum-derived raw materials and degradability after disposal [3,4]. Biopolymers like tannins, lignin, and starch have been proposed to replace the synthetic adhesives used in particleboards [5,6,7]. Zhao et al. used a binder composed of tannin and sucrose to produce particleboards with a density of 800 kg·m^−3^. Thickness swelling ranged from 20 to 23% [7]. Selamat et al. produced particleboards bound with carboxymethyl starch, with densities between 600 and 800 kg·m^−3^ and thickness swelling between 20 and 12% [8]. 

On the other hand, starch is known to be self-foamable, which has led to it being proposed as a natural replacement for some synthetic foam applications [9,10,11]. This implies processing via hot mold baking. During this process, the starch granules gelatinize, forming a viscous paste, and the vaporized water causes the paste to expand. The foam is then dried to allow consolidation [12]. Combination of a starch binder that possesses foaming capability with wood particles seems like a promising strategy to obtain low-density particleboards with good internal cohesion. This approach has been described in our previous work, where a hot-mold was replaced by a hot-press, which is traditionally used among particleboard manufacturers. In this preliminary study, low-density particleboards with densities between 207 and 407 kg·m^−3^ were produced, based on sour cassava starch. Very good internal bond strength and thickness swelling values were obtained, the best performance corresponding to internal bond strength of 0.67 N·mm^−2^, and thickness swelling of 8.7% for a density of 318 kg·m^−3^ [13]. However, in this proof-of-concept work the production process was not optimized, implying excessively long pressing times, in the order of 40 min, which was a major limitation concerning energy-efficiency and productivity. It is therefore relevant to study how these particleboards can be produced using more efficient pressing conditions.

The hot-pressing process is a key step in the production of particleboards, being also the most costly of the whole process. It requires strict control of all pressing parameters in order to ensure the intended physico-mechanical properties of the product and minimize production [14]. These parameters depended on the mat moisture content and the type of binder. High initial mat moisture content implies longer pressing times in order to allow for water vaporization in the center of the board. In the current case, the hot-pressing conditions determine how the starch foam develops, contributing to create air-filled cells in between the wood particles, thus providing low density to the board while ensuring good cohesion and adhesion to the wood particle surfaces.

When heated in water, starch granules become hydrated, swell, and undergo disruption. The crystalline order within the starch is lost and amylose chains leach out from the granule and dissolve in water, causing the solution viscosity to increase significantly and forming a gel [15]. The gelatinization temperature for sour cassava starch is around 88 °C [16]. Above this temperature, water present in the gel vaporizes into trapped air bubbles, causing expansion and formation of the foam cell structure. 

The present work studies how different production parameters affect the properties of low density particleboards bonded with a foamable sour cassava starch formulation. The goal defined by us was to obtain panels with density no higher than about 400 kg·m^−3^ and internal bond strength not lower than 0.35 N·mm^−2^, keeping production time as low as possible.

## 2. Materials and Methods 

### 2.1. Materials

Sour cassava starch was supplied by A Colmeia do Minho S.A. (Seixal, Portugal). Glycerol (99.59%) and propionic acid (99%) were supplied by José Manuel Gomes dos Santos Lda. (Odivelas, Portugal). Chitosan (molecular weight around 300 kDa, degree of deacetylation >85% was purchased from Golden-Shell Pharmaceutical Co. Ltd (Yuhuan, China). Recycled wood particles (eucalyptus, pine, etc.), with moisture content of 4%, used for the manufacture of particleboards were provided by Sonae Industria PCDM (Oliveira do Hospital, Portugal). Pinus pinaster fibers was provided by Valbopan - Fibras de Madeira S.A. (Nazaré, Portugal). 

### 2.2. Preparation of Binder 

The adhesive system was prepared as described in our previous work [13]. Table 1 shows the formulation composition. Initially, sour cassava starch and distilled water were mixed. Chitosan solution, at 5 wt% concentration, was prepared mixing chitosan and propionic acid solution (6 wt%), during 3 h at 60 °C. This solution was added to the starch and water mixture. Finally, Pinus pinaster fibers and glycerol were added and stirring was maintained for 5 min. The binder mixture shows a light brownish color, has a solid content of 38% and a viscosity of 15000 cP (measured in a LVDV-IIIU Brookfield viscometer from Brookfield Engineering Laboratories (Middleboro, MA, USA) in the following conditions: temperature −23.3 °C, spindle-RV7, torque-39.5%, speed-10 RPM).

### 2.3. Scanning Electron Microscopy 

The internal structure of particleboards was observed by Scanning Electron Microscope, using a high resolution (Schottky) Environmental Scanning Electron Microscope with X-ray Microanalysis and Electron Backscattered Diffraction analysis: Quanta 400 FEG ESEM/EDAX Genesis X4M (FEI, Hillsboro, OR, USA). Samples were coated with Au/Pd thin film, by sputtering, using the SPI Module Sputter Coater equipment. The analysis were performed at CEMUP (Centro de Materiais da Universidade do Porto, Porto, Portugal).

### 2.4. Particleboard Production

Wood particles were manually blended with the adhesive system. The adhesive/wood ratio was 1:1 based on weight of solid adhesive content and oven dry wood. Single layer particleboards were hand formed in square aluminium deformable container, with 220 × 220 × 80 mm^3^. A computer-controlled laboratory scale press, equipped with a linear variable displacement transducer (LVDT), pressure transducer and thermocouples, was used to produce the particleboards. 

**Figure 1:**
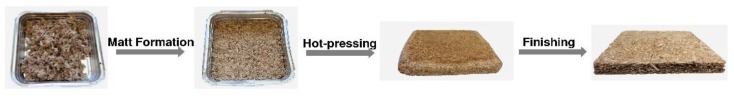
Steps of manufacture process of particleboards bonded with a sour cassava starch, starting with a mixture of wood particles and starch paste and ending with a finished particleboard.

The press bottom platen temperature was set to 190 °C, while the temperature of the top platen was varied between 80 and 190 °C. The adhesive/wood mixture was placed on the bottom platen and pressed to 16 mm thickness for a certain amount of time, designated as “pressing time”. After this, the top platen was raised to 28 mm thickness, and the panel maintained in the press for a duration designated as “hold time”, to allow foam expansion as water in the starch paste vaporizes. The “press cycle time” comprises the pressing and hold times. After pressing, panels were dried at 20 ± 2 °C and relative humidity of 65 ± 5% till constant mass. During this process boards suffer shrinkage due to loss of water, attaining thickness of 16–20 mm depending on the production conditions, which results in different final densities. The mass of wood/binder mixture used to produce the panels was always 425 g, which corresponds to a target density of 318 kg·m^−3^. Four boards were produced for each condition tested. Figure 1 depicts the particleboards manufacturing process. 

Before testing, particleboards surfaces were calibrated by sanding in order to remove irregularities and guarantee flat parallel surfaces. Tested particleboards had a moisture content between 15.1 and 15.5%. 

Density measurements were performed according to EN 323. The samples were square shaped, with side length of 50 mm and thickness between 16 and 20 mm. Density was calculated using the mass and volume of specimen after drying. Four replicates were used for each experiment

Determination of internal bond strength, also known as tensile strength perpendicular to the plane of the board, was performed according to EN 319. The specimen has a square shape with 50 × 50 mm and thickness between 16 and 20 mm. The test pieces are glued to the metal loading block using a hot-melt glue (ethylene vinyl acetate) The specimen is subject to a tensile force at constant speed until rupture occurs. Four replicates were used for each experiment. 

Thickness swelling was determined according to the method described in EN 317. The increase in thickness of a specimen, with 50 × 50 mm^2^ and thickness between 16 and 20 mm, was evaluated after complete immersion in water for 24 h. Four replicates were used for each experiment.

Moisture content was measured according to EN 322. It is the ratio between the weight loss of a sample, dried in an oven at (103 ± 2) °C till constant mass, and the mass of oven dry-board. The specimen had a square shape with 50 × 50 mm^2^ and thickness between 16 and 20 mm. Four replicates were used for each experiment

## 3. Results and Discussion

**Figure 2:**
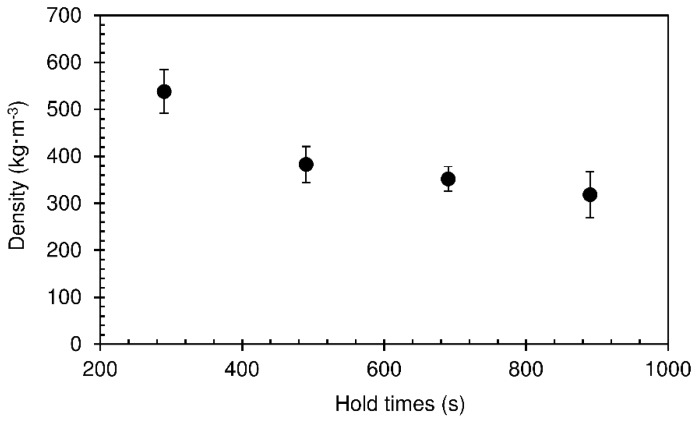
Density of particleboards bonded with sour cassava starch foam for different hold times at 190 °C. The pressing time used was 10 s.

In order to understand the influence of hold time on physico-mechanical properties of particleboards, different times (290–890 s) were tested, with both platens at a temperature of 190 °C. This temperature was chosen because it is typically used in industrial production of particleboards. After being placed on the lower platen, the mat made of wood particles mixed with starch paste was pressed to 16 mm thickness during 10 s, to ensure good particle contact and heat transfer. After this the top platen was lifted to 28 mm and held in this position for the intended hold time, allowing the particle/starch mixture to expand until reaching the top platen. The final densities of the panels for the different hold times are shown in Figure 2.

The obtained densities range between 318 and 538 kg·m^−3^, decreasing with the hold times. The particleboards produced at 290 s did not touch the top platen during expansion. For this reason, these panels had a final thickness of 15.24 mm after being removed from the press (Table 2) and their density is higher than de others. For longer, the panels expanded until reaching the 28 mm limit defined by the top platen. However, when removed from the press, all panels ended up abating as vapour escaped while the starch is still soft, allowing the foam to practically collapse. These panels had a final thickness between 18.31 mm and 20.62 mm (Table 1). The reduction in thickness after abatement is lower for the longer times due to progressive hardening of the starch foam as water vaporizes. This leads to the slight decrease in density observed for hold times between 490 and 890 s.

**Figure 3:**
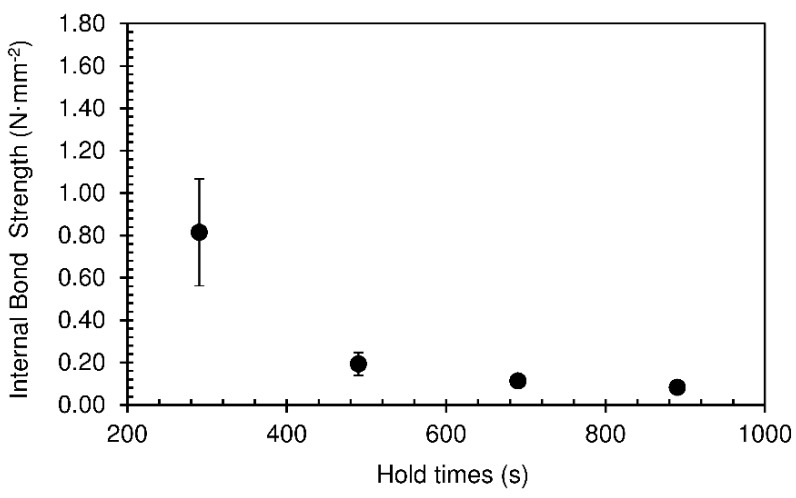
Internal bond strength of particleboards bonded with sour cassava starch foam for different hold times, produced at 190 °C. The pressing time used was 10 s.

The internal bond strength of particleboards indicates the level of cohesion inside the panel, or, in other words, how well particles are bonded together. Figure 3 shows the results of internal bond strength obtained for the particleboards produced. 

The reduction in internal bond strength follows the decrease in density, which was expected since lower density implies lower internal cohesion. However, the very low values obtained for hold time of 490 s and above are actually attributable to rupture of the foam structure after the panel is removed from the press. The large amount of water vapour trapped inside the panel creates significant inner pressure, which causes very fast expansion followed by collapse when it is removed from the confinement of the press platens. This results in bursting of the foam cell walls, creating interior cracks that weaken the panel. 

The relatively high variability observed in internal bond strength for the lower hold time, when the panels display higher strength, is attributable to several factors, like the heterogeneity of the particles, which are obtained from recycled wood, and the inhomogeneity of the adhesive/wood mixing process, which is done manually. This variability, observed also for other test conditions, could be reduced if more replicates were performed, but this would not have an effect on the key conclusions that can be drawn from the results. 

**Figure 4:**
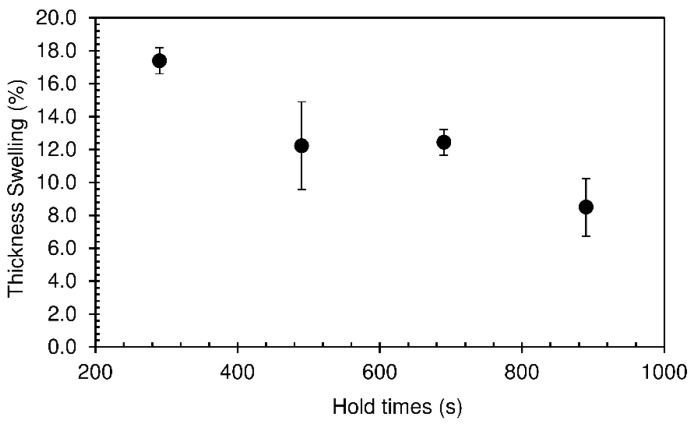
Thickness swelling of particleboards bonded with sour cassava starch produced at different hold times at 190 °C. The pressing time used was 10 s.

The thickness swelling results are shown in Figure 4. As expected, thickness swelling follows the same trend as density. The lower the mass of material per unit volume, the lower will be the amount of water absorbed and thus the lower the swelling. 

According to the European standard EN 312, for non load-bearing boards for use in humid conditions (P3 class), with thickness between 13 and 20 mm, the maximum thickness swelling allowed is 14%. Particleboards produced with longer hold times (490–890 s) meet this requirement. 

**Figure 5:**
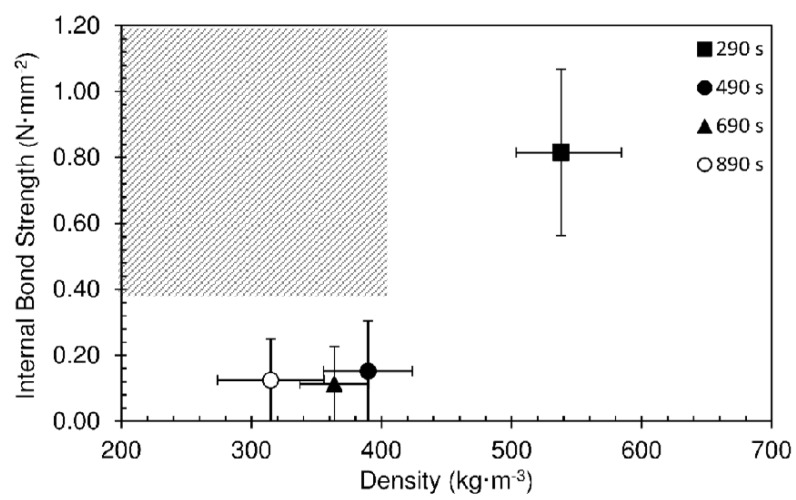
Internal bond strength as function of density for particleboards produced at different hold times and 190 °C. The pressing time used was 10 s.

Figure 5 shows an overview of the internal bond strength as a function of density for the particleboards produced at different hold times. The grey area in the graph represents the goal region of the present work: low density particleboards (density ≤ 400 kg·m^−3^) with good bonding quality (internal bond strength ≥ 0.35 N·mm^−2^). It can be seen that none of the panels produced in these conditions satisfied the objectives. Low density implies low strength and good strength is obtained only with too high density. 

**Figure 6:**
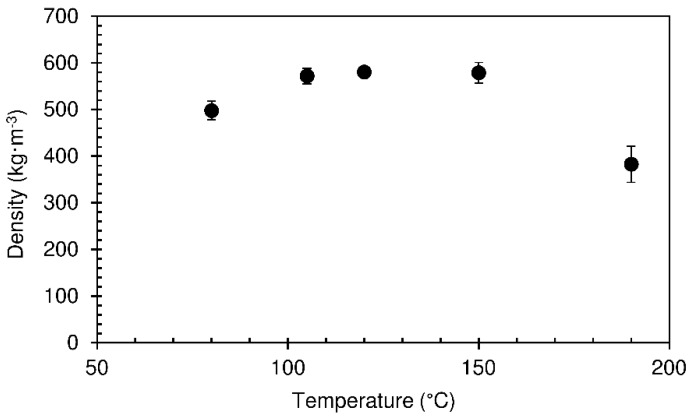
Density of particleboards produced with different top platen temperatures, for a hold time of 490 s. The pressing time used was 10 s.

Considering the previous results, a hold time of 490 s was selected and the effect of decreasing the top platen temperature was studied. The lower platen was kept at 190 °C. The particleboard density results are shown in Figure 6. 

For temperatures up to 150 °C, the density does not change significantly with temperature. On the other hand, for 190 °C it decreased from around 580 to 380 kg·m^−3^. The high densities observed at low temperatures are a result of panel abatement after removal from the press. This occurs due to incomplete foam consolidation. Starch foam is formed as water vapor bubbles are trapped within the viscous paste of gelatinized starch [12]. Gelatinization is a condition for starch’s crystalline double-helix chains to dissociate, breaking up the granules’ structure and forming a waterborne network of hydrogen bonds [17]. Low temperatures in the top platen do not allow for complete starch gelatinization, and a fraction of starch chains remain within the granules. As a consequence, the foam cell walls do not develop the strength needed to hold the structure when the vapour escapes after the panel is removed from the press. At 190 °C, gelatinization within the mixture is complete and the panel undergoes significantly less abatement, allowing for lower final density. 

**Figure 7:**
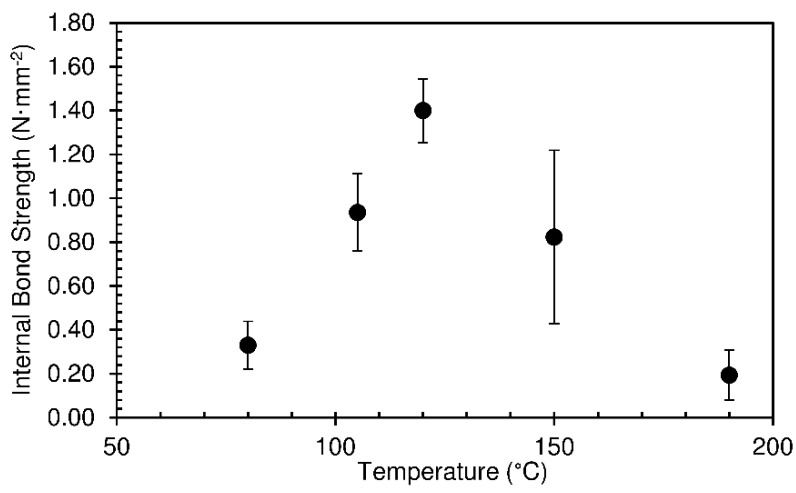
Internal bond of particleboards produced at different top platen temperatures, for hold time of 490 s. The pressing time used was 10 s.

The internal bond strength results are shown in Figure 7. Bond resistance increases very significantly for top platen temperatures between 80 and 120 °C, which is not relatable to the trend observed in the panel densities, which did not change significantly. This strength increase is coherent with a more extensive gelatinization as the mixture is heated more effectively, as discussed above. For 150 °C the measurements showed large variability, indicating that this is a point of instability. For 190 °C, the bond strength decreased to a very low value, which is a consequence of inner bursting of the foam when this panel with a more hardened foam structure was taken out of the press confinement, as mentioned before. 

**Figure 8:**
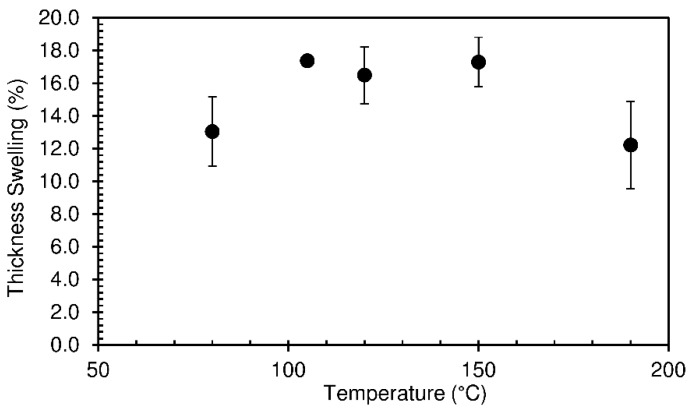
Thickness swelling of particleboards bonded with sour cassava starch foam produced at different pressing temperatures with a hold time of 490 s. The pressing time used was 10 s.

The thickness swelling results are shown in Figure 8. Thickness swelling values vary between 12.2 and 17.4%, being once again relatable to the changes in density. 

**Figure 9:**
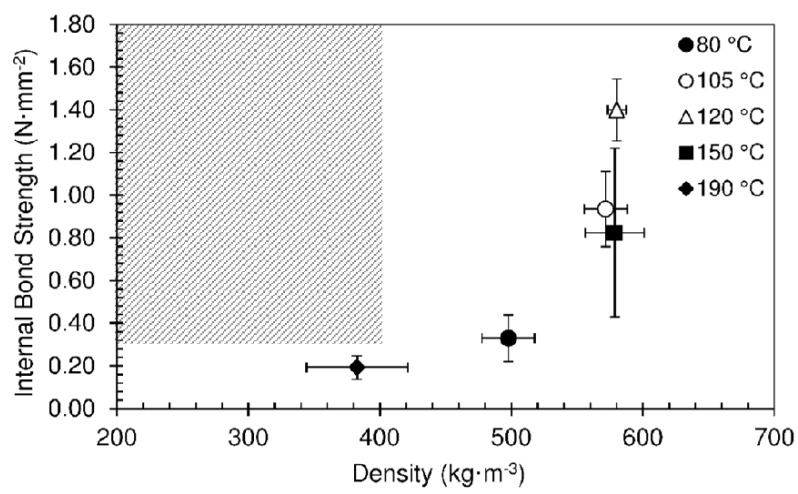
Internal bond strength as function of density for particleboards produced at different temperatures with a hold time of 490 s. The pressing time used was 10 s.

Figure 9 shows an overview of the results obtained for panels produced at different temperatures. Once again, none of the pressing conditions allowed us to obtain particleboards inside the target area. Decreasing the press top platen temperature mostly led to higher final densities, contradicting the intended goal. 

The previous studies indicated that manipulating the hold time and the top platen temperature did not allow attaining the combined density and internal bond strength goal. Since there was evidence that in some conditions complete starch gelatinization may be hindered, a closer look was taken at the temperature inside the particle/starch mat during the panel manufacture process. In addition, the expansion of the mat was monitored by image analysis. 

**Figure 10:**
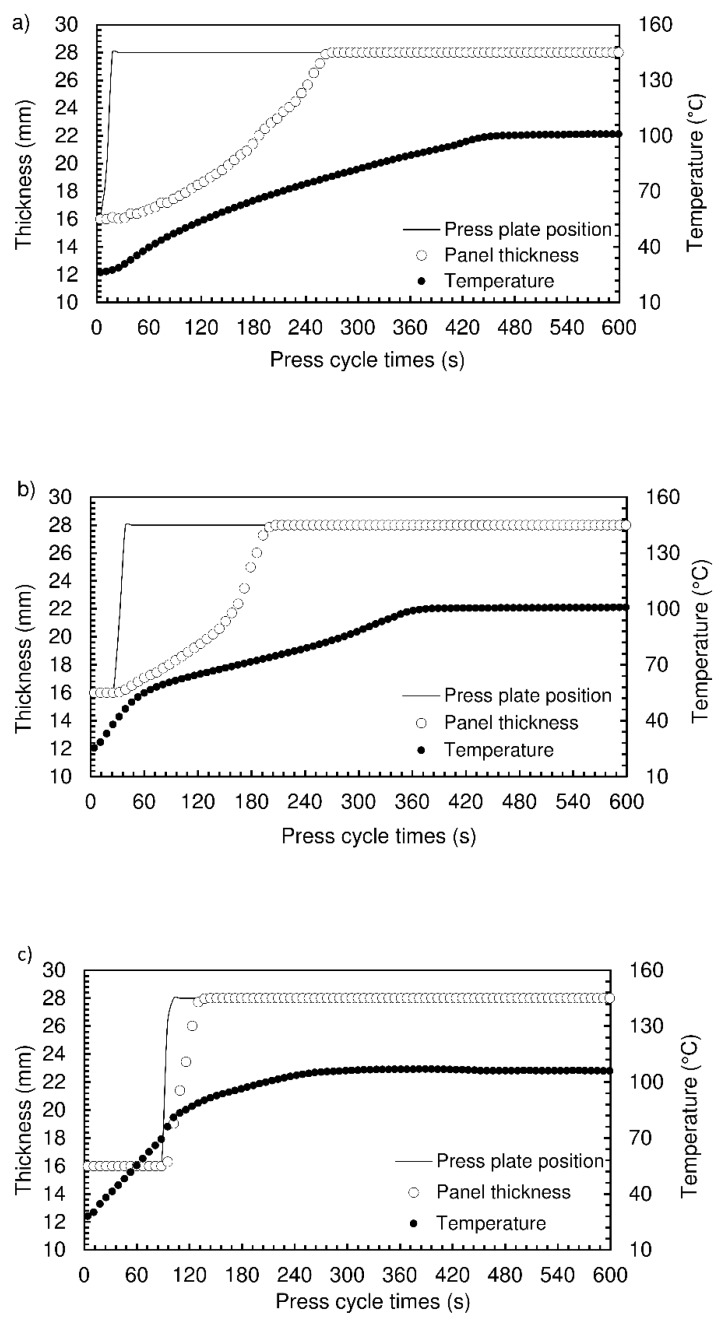

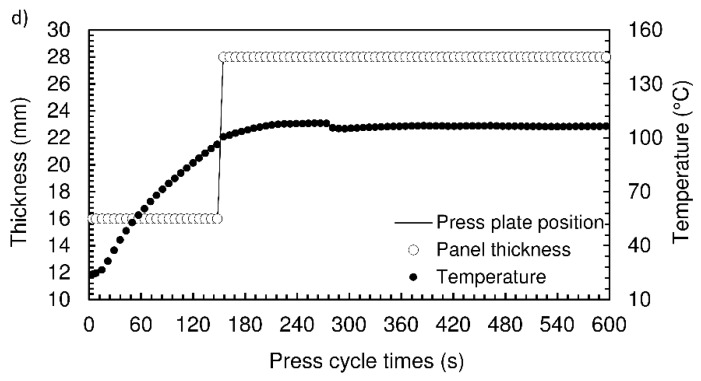
Expansion behavior profile of particleboards bonded with sour cassava starch for different pressing times: 10 s (**a**), 30 s (**b**), 90 s (**c**), and 150 s (**d**). The pressing temperature of experiments was 190 °C.

The top platen temperature was kept at 190 °C, and different pressing times, at which the mat is pressed at 16 mm thickness, were tested: 10 s, 30 s, 90 s, 150 s. The total time in the press was 600 s. Figure 10 shows the results obtained, in terms of mat thickness and inside temperature histories for the pressing times tested. 

**Figure 11:**
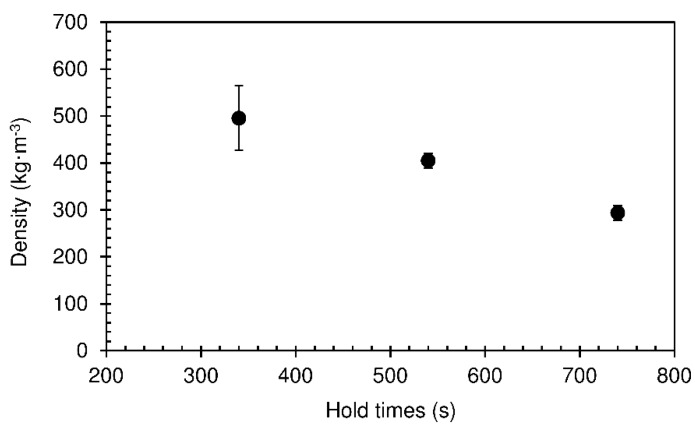
Density of particleboards bonded with sour cassava starch for different hold times at 190 °C. The pressing time used was 60 s.

The temperature inside the mat increases relatively slowly when the pressing time is only 10 s, taking about 450 s to reach a maximum slightly above 100 °C (Figure 10a). This maximum temperature is dictated by the continuous evaporation of liquid water present in the starch paste. The foam expansion is also relatively slow. The mat reaches the top platen only about 250 s after the beginning of the process, corresponding to a hold time of 240 s. As the pressing time is increased, heating inside the mat is accelerated, as well as its expansion. When the pressing time is 150 s, the center of the mat has almost reached the maximum temperature by the end of pressing, and the expansion actually follows the movement of the top platen as soon as it rises (Figure 10d). The pressing time is therefore seen to play an important role, since it corresponds to a highly effective heat transfer process that accelerates water evaporation and foam formation. However, it must be noted that the panels produced at the higher pressing times (90 and 150 s) exhibited internal fractures, associated with the vapour pressure build-up within the mat causing bursting of the foam structure when the top platen was raised. It was therefore decided to try a pressing time of 60 s in the subsequent panel productions, keeping the top platen temperature at 190 °C, and testing different hold times. The density results are shown in Figure 11.

**Figure 12:**
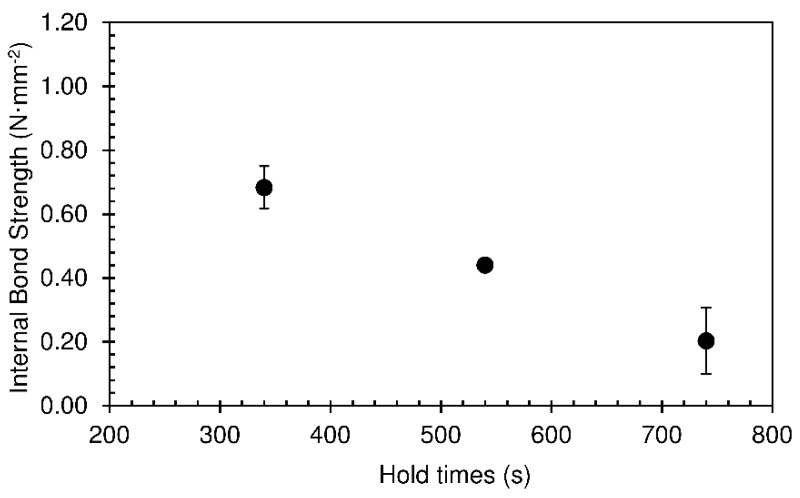
Internal bond of strength particleboards bonded with sour cassava starch for different hold times at 190 °C. The pressing time used was 60 s.

The minimum value of density obtained was 294 kg·m^−3^ for a hold time of 740 s. The density of the panels decreases with the increase in hold time. Longer time in the press implies more water evaporation and hardening of the foam, therefore diminishing abatement when, after removal from the press, trapped vapour escapes through the panel surface. Figure 12 shows that the panels did not undergo internal rupture of the foam structure, since reasonably high values of internal bond strength were obtained. As expected, these followed the same trend as density. 

**Figure 13:**
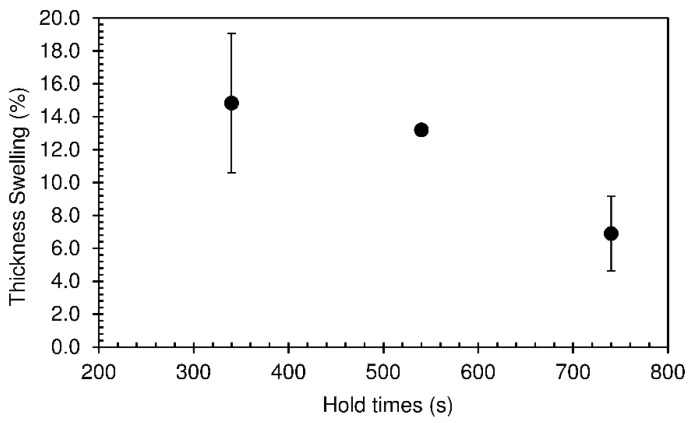
Thickness swelling of particleboards bonded with sour cassava starch for different hold times at 190 °C. The pressing time used was 60 s.

The thickness swelling results are shown in Figure 13. The values obtained ranged between 6.9 and 14.8%. As discussed before, these can be considered quite low when compared with particleboards produced with a bio-based adhesive reported in the literature. 

**Figure 14:**
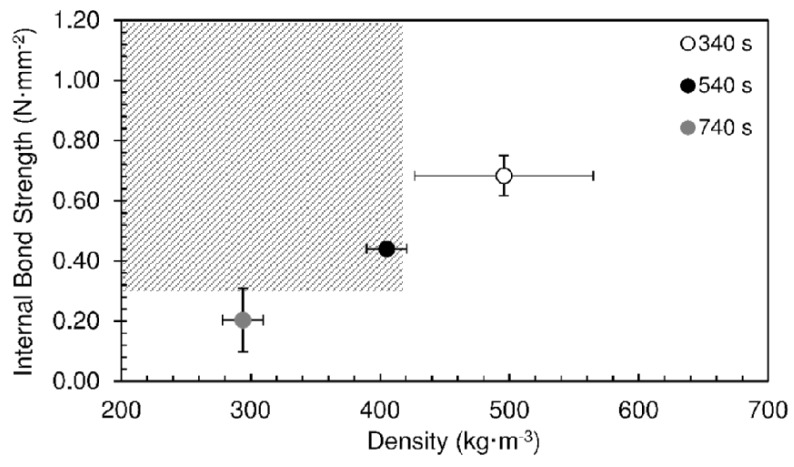
Internal bond strength as function of density for particleboards bonded with sour cassava starch for different hold times at 190 °C. The pressing time used was 60 s.

Figure 14 shows an overview of the results obtained for panels produced with the pressing time of 60 s for different hold times at 190 °C. The work’s goals were attained with 540 s. These operating conditions allowed us to produce particleboards with density of 405 kg·m^−3^, internal bond strength of 0.44 N·mm^−2^, and thickness swelling of 13.2%. It is well known that particleboards produced with bio-based adhesives tend to have lower internal bond strength than those based on conventional urea-formaldehyde resins for the same densities. Zhang et al. produced particleboards using bayberry tannin-based adhesive having internal bond strength obtained of 0.34–0.47 N·mm^−2^, but with densities of 600–718 kg·m^−3^ [18]. Ferreira et al. used a binder system based on spent sulfite liquor and wheat flour, obtaining maximum internal bond strength of 0.46 N·mm^−2^ for boards with a density of 650 kg·m^−3^ [6]. Sulaiman et al. produced boards using epichlorohydrin-modified rice starch as binder. They obtained internal bond strength of about 0.22 N·mm^−2^ for native rice starch, and 0.35–0.39 N·mm^−2^ for modified rice starch, with densities of 600 kg·m^−3^. The best performance corresponded to panels manufactured with epichlorohydrin-modified rice starch combined with urea-formaldehyde resin, yielding internal bond strength of 0.50–0.61 N·mm^−2^ [19]. 

**Figure 15:**
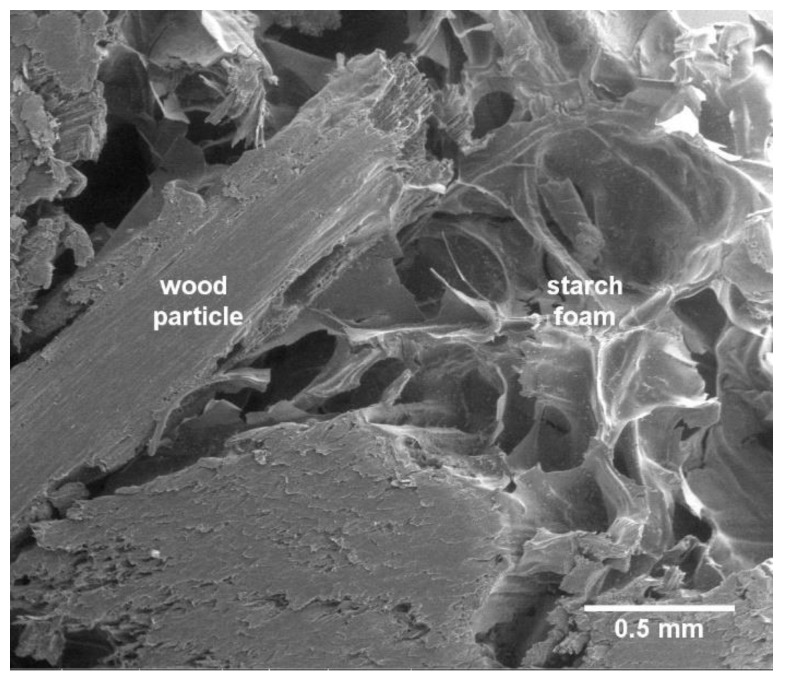
SEM image of the interior of the particleboards showing wood particle surrounding by a starch foam, 100× magnification.

Figure 15 shows a SEM image of the interior of a panel. It is visible that the wood particles are surrounded by starch foam, and an apparently good interphase adhesion exists between the two. The starch foam effectively separates the wood particles, providing the panel’s low density, while providing cohesive strength, as demonstrated by the good internal bond resistance values obtained. Note that, according to European Technical Specification CEN/TS 16368, for lightweight particleboards type LP2 (the most demanding) with thickness between 13 mm and 20 mm, the minimum requirement for internal bond is 0.35 N·mm^−2^. 

## 4. Conclusions

The present work studied the effect of different production parameters on the physico-mechanical properties of low density particleboards bonded with a foamable sour cassava starch formulation. Our aim was to produce totally biosourced panels with density about 400 kg·m^−3^ and internal bond strength higher than 0.35 N·mm^−2^, in the shortest production time possible.

The particleboards produced with hold times ranging from 290 to 890 s had densities between 318 and 538 kg·m^−3^. Density decreased with hold time, however above 490 s the density variation was small. In this case the panels showed very low internal bond strength (<0.20 N·mm^−2^), due to bursting of the internal foam cell structure upon removal from the press, a consequence of the large amount of water vapour entrapped within the panel.

The pressing temperature affects starch gelatinization, and consequently the consolidation of the foam structure. Panels produced at low top platen temperatures (<150 °C) showed high densities as a result of the foam structure abating after vapour escapes when the panel is removed from the press. Starch gelatinization is more effective at 190 °C, and lower densities can be obtained.

Longer pressing times accelerate foam expansion due to more effective heat transfer within the particle mat. Nonetheless, panels manufactured with too long pressing times (90 and 150 s) exhibited internal fractures due to vapour pressure build-up. 

The selected operating parameters were: Pressing time of 60 s, platen temperature of 190 °C, and total hold time of 540 s. Particleboards produced under these conditions had density of 405 kg·m^−3^, internal bond strength of 0.44 N·mm^−2^, and thickness swelling of 13.2%. The internal bond strength obtained is above of the requirements of European Technical Specification CEN/TS 16368, for lightweight particleboards type LP2 (the most demanding) (0.35 N·mm^−2^).

## Figures and Tables

**Table 1:** Formulation of the adhesive system.

Component	Quantity (wt%)
Sour cassava starch	30.5
Distilled water	30.5
Chitosan solution	33.4
Pinus pinaster fibers	2.8
Glycerol	2.8

**Table 2:** Panels thickness measured immediately after removed from the press (results for four specimens).

Hold Times (s)	Thickness of Panels Immediately after Pressing (mm)
290	15.24 ± 0.07
490	18.31 ± 0.54
690	20.62 ± 0.95
890	20.56 ± 0.28
